# Complete Genome Sequence of Citrobacter braakii GW-Imi-1b1, Isolated from Hospital Wastewater in Greifswald, Germany

**DOI:** 10.1128/mra.00143-23

**Published:** 2023-04-18

**Authors:** Dominik Schneider, Aygun Ganbarzade, Sina Post, Daniela Zühlke, Tjorven Hinzke, Jacqueline Hollensteiner, Anja Poehlein, Katharina Riedel, Rolf Daniel

**Affiliations:** a Genomic and Applied Microbiology and Göttingen Genomics Laboratory, Institute of Microbiology and Genetics, Georg-August University of Göttingen, Göttingen, Germany; b Department of Microbial Physiology and Molecular Biology, Institute of Microbiology, University of Greifswald, Greifswald, Germany; DOE Joint Genome Institute

## Abstract

The imipenem-resistant Citrobacter braakii strain GW-Imi-1b1 was isolated from a hospital wastewater sample in Greifswald, Germany. The genome comprises one chromosome (5.09 Mb), one prophage (41.9 kb), and 13 plasmids (2 to 140.9 kb). The genome harbors 5,322 coding sequences, shows a high potential for genomic mobility, and includes genes encoding proteins for multiple drug resistances.

## ANNOUNCEMENT

*Citrobacter* strains thrive in soil, water, and gut microbiota of humans and animals. They cause nosocomial infections and show various degrees of antibiotic resistance (1). Citrobacter braakii GW-Imi-1b1 is an aerobic Gram-negative, rod-shaped, motile, imipenem-resistant bacterium that was isolated from wastewater from the Greifswald University Hospital in October 2020. It grew in the inhibition zone of an imipenem-sensitive Citrobacter portucalensis isolate (10-μg imipinem disc; Bestbion dx GmbH, Cologne, Germany) on Mueller-Hinton agar (Carl Roth GmbH & Co. KG, Karlsruhe, Germany).

The manufacturer’s recommendations were followed unless otherwise stated. Genomic DNA of C. braakii GW-Imi-1b1 was extracted using the MasterPure complete DNA purification kit (Epicentre, Madison, WI, USA), from a strain that had been isolated by repeated streaking and regrowth in 10 mL Mueller-Hinton broth (Carl Roth GmbH & Co.) at 37°C. Illumina paired-end sequencing libraries were prepared with the Nextera XT DNA sample preparation kit (Illumina, San Diego, CA, USA) and sequenced using a MiSeq system and reagent kit v3 (2 × 300 cycles). Nanopore sequencing library preparation was performed with 1.5 μg high-molecular-weight DNA, a ligation sequencing kit 1D (SQK-LSK109), and the native barcode expansion kit (EXP-NBD104). Sequencing was performed for 72 h using a MinION Mk1B system with a SpotON R9.4.1 flow cell (Oxford Nanopore Technologies, Oxford, UK) and MinKNOW v22.05.5 and Guppy v6.2.1 (hac mode) software.

**FIG 1:**
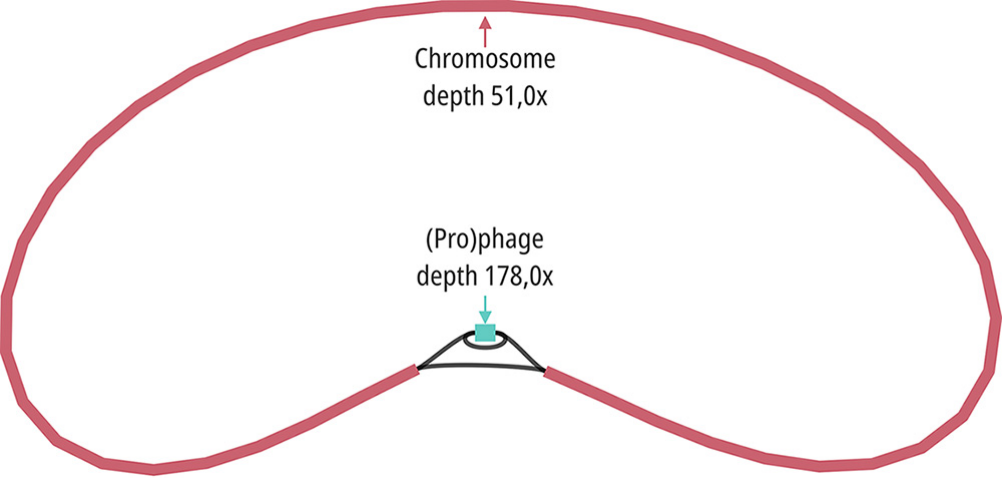
Flye assembly graph of the Citrobacter braakii GW-Imi-1b1 chromosome (red) and phage (blue), including sequence depths (MinION long reads), created with Bandage v0.8.1 (20). The assembly graph and coverage depths (phage/chromosome ratio, 3.5) suggest co-occurrence of the prophage, as well as extrachromosomal prophage or phage particles within the Citrobacter braakii GW-Imi-1b1 culture.

Default settings were used for all software unless otherwise specified. Illumina and Nanopore reads were quality filtered using fastp v0.23.2 (2). In addition, Cutadapt v3.2, followed by Bowtie2 v2.3.5.1 (3), was used on short reads to remove potential adapter and phiX (GenBank accession number NC_001422) leftovers. Furthermore, long reads were treated with Porechop v0.2.4 (https://github.com/rrwick/Porechop) and adjusted to 100× coverage with Filtlong v0.2.1 (https://github.com/rrwick/Filtlong), and chimeric reads were removed with unicycler_scrub of Unicycler v0.4.9 (4). Sequencing details are shown in Table 1. Flye v2.9.1-b1780 (5) assembled long reads into chromosome and phage. The Flye assembly graph and coverage indicate co-occurrence of the phage as an integrated prophage as well as extrachromosomal prophage or phage particles within the culture of C. braakii GW-Imi-1b1. Unicycler v0.5.0 (4), employing the assembly graph of Flye, was used for *de novo* hybrid assembly of plasmids (confirmed by BLAST) (Fig. 1). Finally, all genome sequences were polished with BWA v0.7.17-r1188 (6) and Polypolish v0.5.0 (7), employing quality-filtered short reads.

**TABLE 1:** Sequencing and assembly statistics of Citrobacter braakii GW-Imi-1b1 and characteristics of the genomic elements

Genomic element	Size (bp)	No. of chromosomes	No. of extrachromosomal elements	No. of MiSeq raw reads	No. of MiSeq processed reads	No. of Nanopore raw reads	No. of Nanopore processed reads	Mean coverage (×)^*a*^	Long-read *N*_50_ (bp)	Long-read *N*_90_ (bp)	Genome assembly *N*_50_ (bp)	Genome assembly *N*_90_ (bp)	GC content (%)	Mean coverage (×)		Copy no.			No. of coding sequences	No. of insertion sequences/transposases	No. of antibiotic resistance genes
														Short reads	Long reads	Short reads	Long reads	Avg^*b*^			
C. braakii GW-Imi-1b1 genome	5,430,182	1	14	2,851,734	2,753,172	170,754	42,449	106.6	57,083	17,772	5,092,943	5,092,943									
Chromosome	5,092,943												51.81	16.8	51.3	1	1	1	4,898	161	18
Phage	41,923												52.86	21.1	217.5	1	4	3	73	0	0
Plasmid 1	140,871												50.12	174	363.8	10	7	9	154	29	0
Plasmid 2	39,337												46.19	1,099	886.2	66	17	41	57	3	0
Plasmid 3	28,609												56.12	1,357	1,143.4	81	22	52	36	4	4
Plasmid 4	24,320												60.73	1,759	1,151	105	22	64	28	3	4
Plasmid 5	14,211												55.64	5,285.5	3,608.9	315	70	193	17	6	1
Plasmid 6	11,263												46.43	2,546.1	1,464.5	152	29	90	13	1	0
Plasmid 7	10,155												47	2,379.1	951.6	142	19	80	15	0	0
Plasmid 8	5,388												44.52	7,748.5	1,546.2	462	30	246	6	0	0
Plasmid 9	5,310												46.08	7,729.7	1,902.9	461	37	249	7	0	0
Plasmid 10	5,198												46.08	13,917.4	2,138.6	830	42	436	4	0	0
Plasmid 11	4,593												34.99	9,527.2	1,952.4	568	38	303	7	0	0
Plasmid 12	4,127												45.41	4,465.9	403.7	266	8	137	5	0	0
Plasmid 13	1,934												51.29	2,1829.3	28.5	1,302	1	651	2	0	0

a Average of short- and long-read mean coverages estimated by Qualimap with processed reads.

b Average of short- and long-read copy numbers estimated based on one chromosome per cell.

The genome contains one circular chromosome (5.1 Mbp), one circular (pro)phage (41.9 kb [see Fig. S1 at https://figshare.com/articles/figure/Supplementary_Figure_1/22133567]), and 13 circular plasmids (0.2 to 140.9 kb). The coverages of all genetic entities were calculated with Qualimap v2.2.2 (8) using Bowtie2 v2.3.5.1 (3) and minimap2 v2.24-r1122 (9). Annotation was performed with Prokka v1.14.5 (10) using additional hidden Markov model (HMM) databases (PGAP v10.0 [11], Pfam v35 [12], pVOG v20171012 [13], and Resfams v1.2 [14]) and HMMer v3.3.2 (15), pharokka (16), and PHASTER (17), and results are summarized in Table 1. The taxonomic classification of the chromosome was performed with GTDB-Tk v2.1.0 (18), and the nearest relative is Citrobacter braakii ASM207534v1 (GenBank accession number GCF_002075345.1), with a FastANI (19) similarity of 98.62% (see Table S1 at https://figshare.com/articles/journal_contribution/Supplementary_Table_S1/21842058). Genome analysis with Resfams revealed the presence of 27 genes involved in antibiotic resistance, including resistance to β-lactams, aminoglycosides, quinolones, macrolides, nitroimidazoles, streptomycin, and chloramphenicol and efflux pumps (see Table S2 at https://figshare.com/articles/journal_contribution/Supplementary_Table_S2/21857355).

### Data availability.

The assembled genomic entities (chromosome, phage, and plasmids), nucleotide and amino acid sequences, and GenBank records are accessible via Figshare (https://figshare.com/collections/Citrobacter_braakii_GW-Imi-1b1/6440603). The whole-genome project of Citrobacter braakii GW-Imi-1b1 has been deposited in GenBank under the accession numbers CP115723 (chromosome), CP115737 (phage), CP115724 (plasmid 1), CP115729 (plasmid 2), CP115730 (plasmid 3), CP115731 (plasmid 4), CP115732 (plasmid 5), CP115733 (plasmid 6), CP115734 (plasmid 7), CP115735 (plasmid 8), CP115736 (plasmid 9), CP115725 (plasmid 10), CP115726 (plasmid 11), CP115727 (plasmid 12), and CP115728 (plasmid 13), with BioProject accession number PRJNA524094 and SRA accession numbers SRR23033312 (Oxford Nanopore Technologies MinION reads) and SRR23033313 (Illumina MiSeq reads).
